# Lack of insulin resistance in response to streptozotocin treatment in neuronal SH-SY5Y cell line

**DOI:** 10.1007/s00702-019-02118-5

**Published:** 2019-12-19

**Authors:** Fruzsina Bagaméry, Kamilla Varga, Kitti Kecsmár, István Vincze, Éva Szökő, Tamás Tábi

**Affiliations:** Department of Pharmacodynamics, Semmelweis University, Nagyvárad tér 4, Budapest, 1089 Hungary

**Keywords:** Glycogen synthase kinase-3, Insulin resistance, Neurodegeneration, SH-SY5Y cell line, Streptozotocin

## Abstract

Recently, it is suggested that brain insulin resistance may contribute to the development of Alzheimer’s disease; therefore, there is a high interest in its investigation. Streptozotocin (STZ) is often used to induce dysregulation of glucose and insulin metabolism in animal and cell culture models. Alteration in insulin sensitivity however, has not yet been assessed in neuronal cells after STZ treatment. We aimed at studying the concentration dependence of the protective effect of insulin on STZ-induced damage using SH-SY5Y cell line. Cells were treated with STZ and cell viability was assessed by resazurin reduction and lactate dehydrogenase release assays. Low serum (LS) medium was used as control damage. The effect of various concentrations (30, 100, 300, 1000 nM) of insulin was studied on cell viability and glycogen synthase kinase-3 (GSK-3) phosphorylation, an indicator of insulin signaling. STZ induced dose- and time-dependent cytotoxicity, its 1 mM concentration exerted a low, gradually developing damage. The cytoprotective effect of insulin was demonstrated in both STZ and LS groups. Its maximal effect was lower in the STZ-treated cells; however, its effective concentration remained largely unaltered. Insulin-induced GSK-3 phosphorylation was similar in the STZ- and LS-treated cells suggesting unchanged insulin signaling. Our present results indicate that STZ does not induce significant impairment in insulin sensitivity in SH-SY5Y cells, thus in this cell line it is not a good tool for studying the role of insulin resistance in neurodegeneration and to examine protective agents acting by improving insulin signaling.

## Introduction

Alzheimer’s disease is a common neurological disorder worldwide and its prevalence is rapidly increasing. Neuropathologically the disorder can be characterized by accumulation of extracellular amyloid-β (Aβ) plaques and intracellular neurofibrillary tangles that consist of aggregates of hyperphosphorylated tau protein. The neuronal damage leads to complete loss of autonomy, which is a tremendous problem for the patients, their caregivers, and the healthcare system. There is no available medical therapy that either slows or stops the progression of the neural damage (Yilmaz 2015); therefore the development of an appropriate, neuroprotective therapy is crucial and intensively investigated.

Insulin in the central nervous system mediates and controls multiple actions, including metabolic activity, eating behavior, neuronal survival, cognitive functions, especially memory and learning (Kullmann et al. 2016; Santiago and Hallschmid 2019). Insulin signaling in the brain is complex. Similarly to the periphery, the major signaling cascade is the phosphatidylinositol-3 kinase (PI3K)–protein kinase B (Akt/PKB) pathway that, among others, is responsible for phosphorylation and inactivation of glycogen synthase kinase-3 (GSK-3). Increased activity of GSK-3 has been associated with several disorders like Alzheimer’s disease, type 2 diabetes mellitus, some cancers, and other neurological disorders (for review see Salkovic-Petrisic and Hoyer 2007). In case of Alzheimer’s disease GSK-3α is involved in the regulation of amyloid processing (Phiel et al. 2003) and insulin was demonstrated facilitating its non-amyloidogenic pathway (Solano et al. 2000). β-Isoform of the enzyme was shown to be identical to tau protein kinase I and thus is involved in tau phosphorylation and formation of neurofibrillary tangles (Leroy et al. 2002; Ishiguro et al. 1993).

Recently, development of insulin resistance in the brain of Alzheimer’s disease patients and its contribution to neurodegeneration was suggested (Frolich et al. 1998; Hoyer 2004; de la Monte and Wands 2005; Steen et al. 2005; Liu et al. 2011). Impaired downstream signaling of insulin may result in overactivation of GSK-3, and thus can participate in Alzheimer’s disease pathology (Hoyer et al. 1994; de la Monte 2012; Takeda et al. 2011; Salkovic-Petrisic and Hoyer 2007) and the development of dementia (Santiago and Hallschmid 2019; Kullmann et al. 2016; Talbot 2013, 2014). Hence, better understanding of the contribution of impaired insulin receptor signaling to the neurodegeneration in Alzheimer’s disease may open the way to the development of neuroprotective therapies.

Streptozotocin (STZ) is one of the most well-established diabetogenic agents in the research of diabetes mellitus (Radenkovic et al. 2016). It is a glucosamine-nitrosourea compound that is produced by *Streptomyces achromogenes* and in animal models it is widely applied for induction of type 1 diabetes. Injection of a high dose of STZ exerts strong cytotoxic effect on GLUT-2 expressing pancreatic beta cells resulting in loss of insulin secretion (Wu and Yan 2015; Like and Rossini 1976). In lower dose, it is also used for induction of type II diabetes (Wang and Gleichmann 1998; Reaven and Ho 1991). Moreover, Wang et al. (2014) reported accelerated brain aging, hippocampal atrophy, Aβ aggregation, and loss of synaptic connections in STZ-induced diabetic animals.

Local administration of STZ to the brain was shown to impair glucose metabolism (Hellweg et al. 1992; Hoyer 1998) and induce an insulin-resistant brain state, and therefore now is widely used as an animal model for Alzheimer’s disease (Steen et al. 2005; Salkovic-Petrisic et al. 2006; Grunblatt et al. 2007; Agrawal et al. 2011). Hyperactivation of GSK-3β and decreased levels of some brain-specific glucose transporters were reported in intracerebroventricular STZ-treated animals (Deng et al. 2009; Salkovic-Petrisic et al. 2014; Rajasekar et al. 2017). Changes in brain morphology relevant to Alzheimer’s disease, i.e. decreased hippocampal volume and astrogliosis were also detected and accompanied by cognitive decline (Rostami et al. 2017; Shoham et al. 2003), cholinergic impairment (Hellweg et al. 1992), oxidative stress (Deshmukh et al. 2016), and formation of hyperphosphorylated tau protein (Grunblatt et al. 2007).

STZ is also used in in vitro experiments aiming at modeling cellular processes characteristic for Alzheimer’s disease and its potential therapeutic measures (Plaschke and Kopitz 2015; Rajasekar et al. 2016; Guo et al. 2016). STZ exerted dose-dependent cytotoxicity on several neuronal cell types (Plaschke and Kopitz 2015; Isaev et al. 2018; Genrikhs et al. 2017) leading to depolarization of mitochondrial membrane (Genrikhs et al. 2017; Biswas et al. 2017), oxidative stress (Rajasekar et al. 2014), increased apoptosis (Rajasekar et al. 2014; Biswas et al. 2016, 2017), increased tau protein phosphorylation (Biswas et al. 2016), decreased glucose uptake (Biswas et al. 2016, 2017) and expression of GLUTs (Biswas et al. 2017; Sun et al. 2018), reduced expression of insulin receptor substrate-1 (IRS-1) (Wang et al. 2011), and decreased levels of phosphorylated GSK-3 (Plaschke and Kopitz 2015; Rajasekar et al. 2014). The protective effect of insulin against STZ-induced impaired cell viability was observed as well (Genrikhs et al. 2017; Rajasekar et al. 2014).

Alteration in insulin sensitivity, however, has not yet been assessed in the in vitro STZ model, thus the development of neuronal insulin resistance remains to be clarified. In the present work, we aimed at studying the concentration dependence of the protective effect of insulin on STZ-induced damage using SH-SY5Y human neuroblastoma cell line. To minimize the impact of unknown insulin content of fetal bovine serum (FBS) (Burnouf et al. 2016) in culture medium, low (1%) serum (LS) condition was applied and used as positive control.

## Materials and methods

### Materials

Dulbecco’s Modified Eagle Medium/Nutrient Mixture F-12 (DMEM/F12) and FBS were purchased from Corning (Tewksbury, MA, USA) and Biosera (Nuaille, France), respectively. Stable glutamine and Minimum Essential Medium non-essential amino acids solutions were obtained from Pan Biotech (Aidenbach, Germany). Insulin, resazurin based cell viability kit (TOX-8), Triton X-100, phosphatase inhibitor cocktail 2, and western blot reagents and buffer components were purchased from Sigma (St. Louis, MO, USA). CytoTox-ONE LDH Assay kit was supplied by Promega (Madison, WI, USA). STZ was obtained from Cayman Chemical Company (Ann Arbor, MI, USA) and was dissolved freshly in citrate buffer (0.1 M, pH 4.5) immediately before adding to the medium. DuoSet IC, Phospho-GSK-3α/β (S21/S9) and anti-GAPDH specific mouse monoclonal antibodies were provided by R&D Systems GmbH (Wiesbaden, Germany).

### Cell culture and treatment

Human neuroblastoma SH-SY5Y cells (ECACC, UK) were cultured up to 72 h in DMEM/F12 containing 10% FBS, 1% stable glutamine, and antibiotics.

For the cell viability assays, the cells were seeded to 24-well plates (2 × 10^4^ cell/well). Twenty four h later the medium was changed to LS one containing 1% FBS and treated by various concentrations of STZ (0.3, 1, 3, 5, 10 mM). In some experiments, the cells were simultaneously treated by insulin (30, 100, 300, 1000 nM). The medium was refreshed daily and insulin treatment was repeated alongside.

For western blot and ELISA assays, cells were seeded to 10 cm Petri dishes (6 × 10^5^ cells/dish) and after reaching approximately 80% confluency, the cells were treated by LS medium and STZ as described above. Twenty four h later, the cells were treated with insulin (30, 100, 300, 1000 nM) for 30 min, and then harvested using lysis buffer (1 mM EDTA, 0.5% Triton X-100, 6 M urea in PBS) containing phosphatase inhibitor cocktail 2.

### Resazurin reduction cell viability assay

Resazurin reduction was assessed according to the manufacturer’s instructions. Briefly, after 24, 48, 72 h treatment, the medium was replaced by fresh medium containing 10% resazurin solution (0.15 mg/ml in PBS) and incubated for 4 h at 37 °C, and the fluorescence of resorufin formed was measured by Fluoroskan Ascent FL microplate fluorimeter (Thermo Fisher Scientific, Waltham, MA, USA) at 530/590 nm.

### Lactate dehydrogenase (LDH) release cytotoxicity assay

Lactate dehydrogenase release was assessed according to the manufacturer’s instructions. After 24, 48, 72 h, media were collected before refreshment. On the last day, cells were lysed using 1% Triton X-100 to determine the intracellular LDH content. LDH activity in the media was assessed according to the manufacturer’s instructions and the fluorescent product was measured with microplate fluorimeter at 530/590 nm.

### Western blot analysis

Total protein concentration of cell lysates was determined by Bradford’s method (Bradford 1976). After denaturation by heating at 95 °C for 5 min in Laemmli buffer [0.1% 2-mercaptoethanol, 0.0005% bromophenol blue, 10% glycerol, 2% SDS, 63 mM Tris–HCl (pH 6.8)], samples were separated in 10% SDS polyacrylamide gels and then transferred onto PVDF membranes. Membranes were then blocked with 5% non-fat dry milk dissolved in Tris-buffered saline containing 0.1% Tween 20 (TBST) for 1 h and then probed with and 0.5 µg/ml anti-GSK, 0.25 µg/ml biotinylated anti-phospho-GSK-3, and 0.25 µg/ml anti-GAPDH antibodies in 5% non-fat dry milk solution overnight at 4 °C. The membranes were washed with TBST thrice for 10 min and then incubated with horseradish peroxidase conjugated to secondary antibodies or streptavidin for 1 h. Specific proteins were detected on autoradiography films using enhanced chemiluminescence reagent.

### ELISA measurement

GSK-3 phosphorylation was measured according to the manufacturer’s instructions with some modifications. Briefly, 100 µl cell lysate was added to the ELISA plate and incubated overnight at 4 °C. After washing, the plate was probed by detection antibody for 4 h at room temperature. The results were corrected by the protein content of the lysates.

### Statistics

Data are expressed as mean ± standard deviation of at least three parallel measurements. One-way ANOVA was used for data analysis followed by Dunnett’s post hoc test for multiple comparisons. Concentration-response curves were constructed by non-linear regression method and their parameters were compared by *F* test. Corrected *p* < 0.05 was considered statistically significant. Data were analyzed by Prism 6.0 software (GraphPad Software Inc., La Jolla, CA, USA).

## Results

### Effect of STZ on cell viability

The cytotoxic effect of STZ compared to LS condition was studied on SH-SY5Y cells. Cells were treated with 0.3, 1, 3, 5, 10 mM STZ in LS medium and cell viability was measured by resazurin reduction and LDH release assays after 24, 48, and 72 h.

**Fig. 1:**
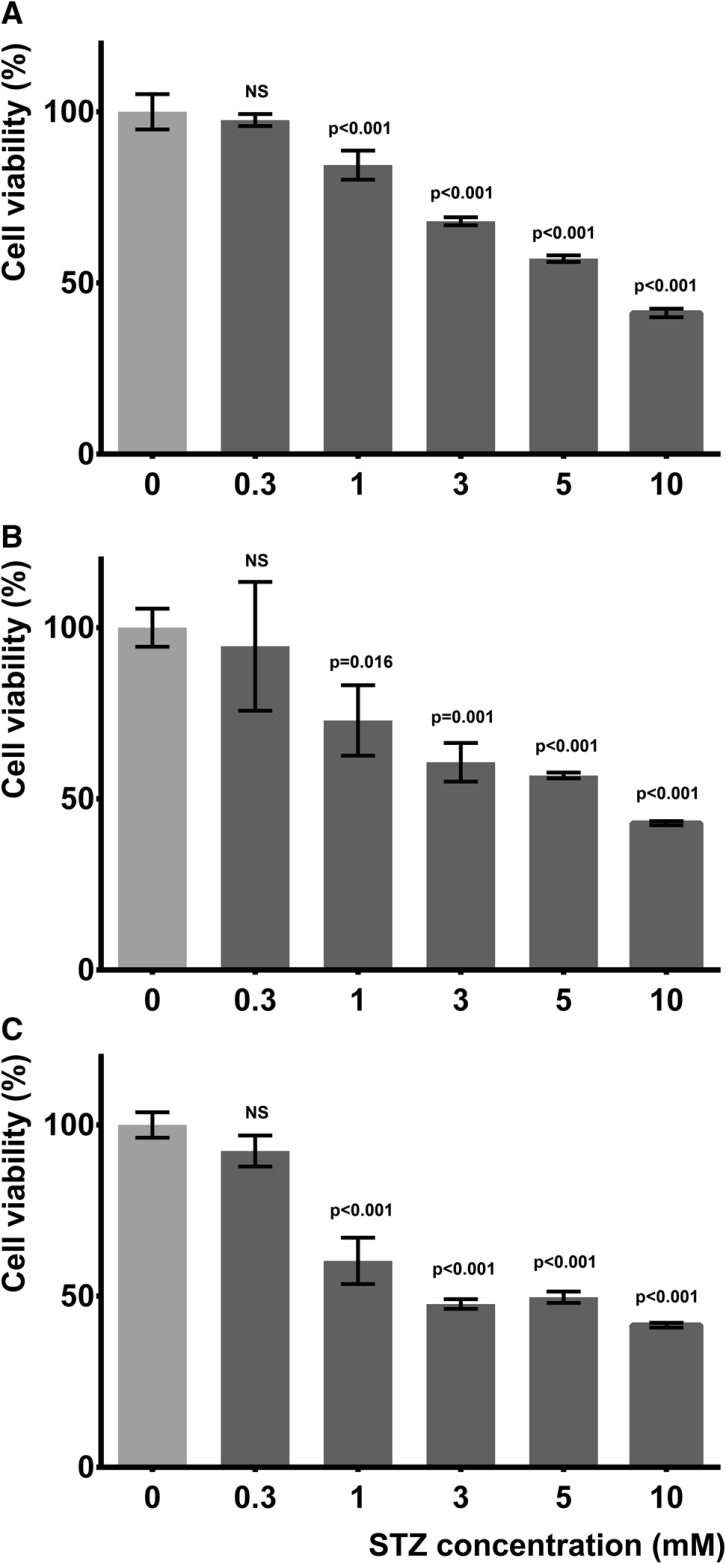
STZ dose and time dependently decreased metabolic activity of SH-SY5Y cells. LS condition was considered as control damage, which impaired the resazurin reducing activity by 37.6%, 42.3%, 44.1% after 1, 2, and 3 days, respectively. Effect of various concentrations (0.3, 1, 3, 5 and 10 mM) of STZ on resazurin reducing activity of cells compared to LS condition after 1, 2, and 3 days (**a**–**c**, respectively) is shown. *P* values compared to respective LS condition (0 mM STZ) are indicated, *NS* non-significant

Low concentration (0.3 mM) STZ caused only mild, non-significant reduction in the number of metabolically active cells, while higher concentrations (3, 5, 10 mM) considerably decreased resazurin reducing activity even acutely after 24 h. One mM concentration of STZ caused mild but significant acute cytotoxicity that increased gradually on the second and third days, so it better models the progressive neurodegenerative processes (Fig. 1).

**Fig. 2:**
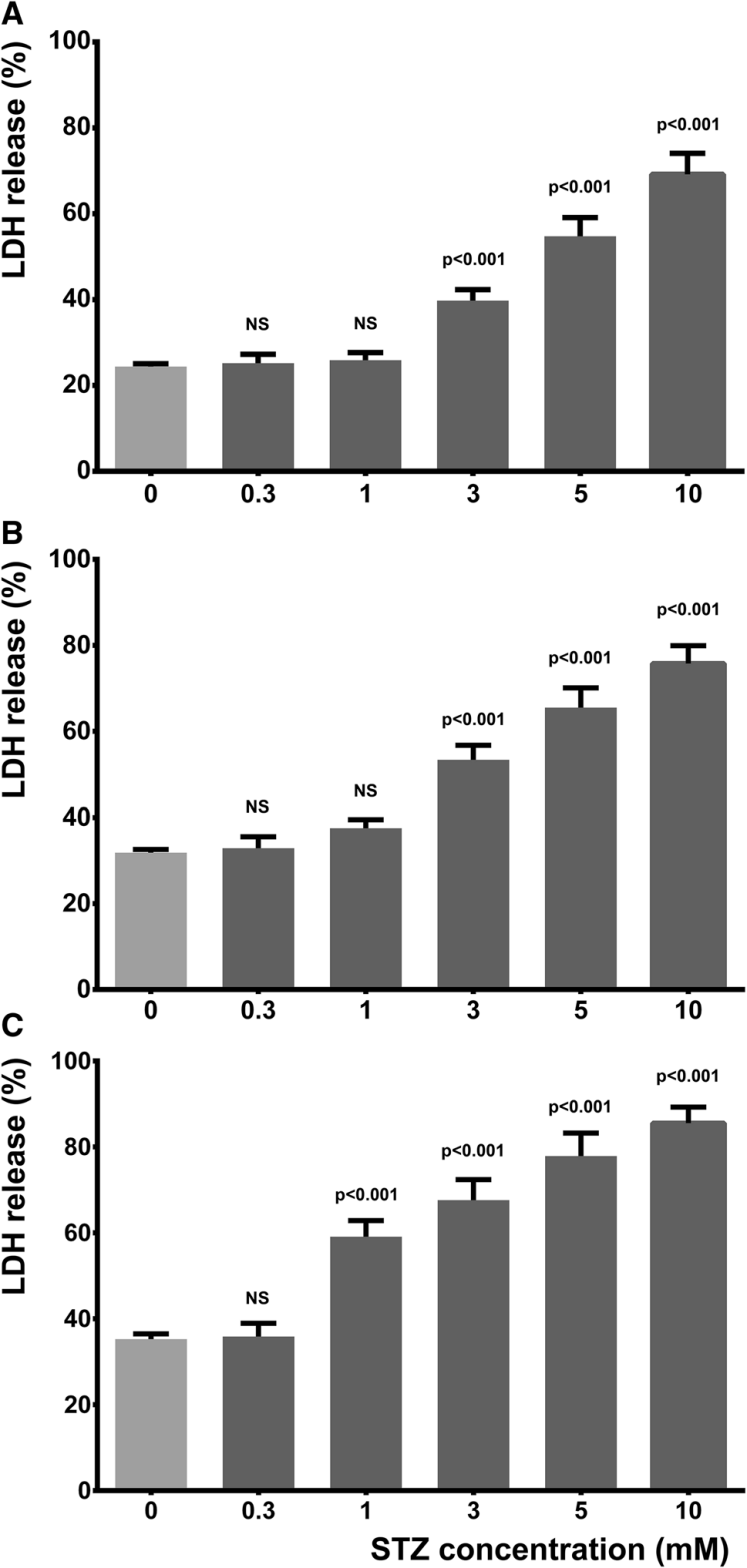
STZ dose and time dependently compromised plasma membrane integrity of SH-SY5Y cells. LS condition was considered as control damage. Effect of various concentrations (0.3, 1, 3, 5 and 10 mM) of STZ on LDH release after 1, 2, and 3 days (**a**–**c**, respectively) is shown. *P* values compared to respective LS condition (0 mM STZ) are indicated, *NS* non-significant

LDH release test was used to confirm the results of resazurin reduction assay. Membrane integrity of cells was slightly compromised in LS medium and in the presence of 0.3 mM STZ for up to 3 days. One mM STZ caused gradual increase in the fraction of cells with membrane damage that became statistically significant after 3 days. In case of higher concentrations of STZ (3–10 mM), however similarly to the loss of metabolic activity, membrane integrity was significantly compromised even within 24 h (Fig. 2).

In the further experiments, thus 1 mM STZ was used to induce gradual neuronal damage.

### Cytoprotective effect of insulin on LS- and STZ-treated cells

**Fig. 3:**
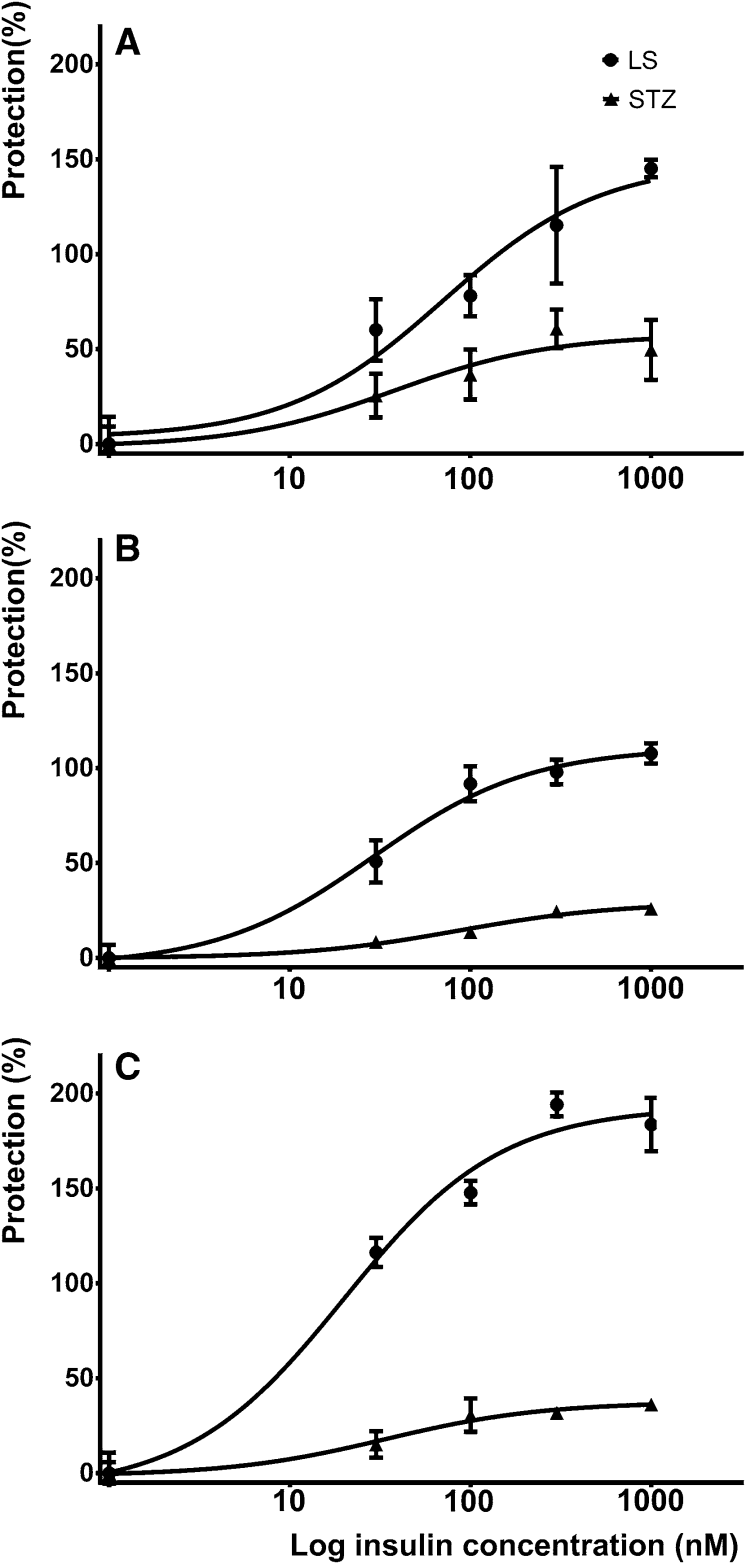
Insulin dose and time dependently improved metabolic activity in LS- and 1 mM STZ-treated cells. Effect of various concentrations (30, 100, 300, 1000 nM) of insulin on resazurin reducing activity of LS- and STZ-treated cells after 1, 2, and 3 days (**a**–**c**, respectively) is shown. Data are expressed as percent protection against damage

The protective effect of various concentrations (30, 100, 300, 1000 nM) of insulin was studied for 1–3 days on cells cultured in LS medium in the presence or absence of 1 mM STZ. Insulin dose dependently attenuated cellular damage that was detected by resazurin reduction assay. The damage caused by LS condition was completely reversed at all time points, furthermore on day 3 the number of viable cells was almost doubled. In case of STZ treatment, insulin showed only partial protection, cell viability was plateaued between 25–50% of the control group (Fig. 3). The insulin concentrations inducing half maximal protection, however, were not significantly different (Table 1) in the two groups.

**Table 1:** Comparison of estimates of *E*_max_ and EC50 of the protective concentration-response curve of insulin in LS- and STZ-treated cells measured by resazurin reduction assay

	*E*_max_					EC50				
	LS		STZ		*P*	LS		STZ		*P*
	Estimates	95% CI	Estimates	95% CI		Estimates	95% CI	Estimates	95% CI	
Day 1	148.3	121.30–175.30	57.5	42.59–72.39	0.0015	70.9	30.08–167.30	37.5	9.461–148.30	0.4715
Day 2	110.1	102.00–119.70	29.1	25.41–32.72	0.0006	28.9	17.51–47.93	86.7	50.04–150.2	0.0843
Day 3	193.1	179.8–206.4	37.4	30.80–43.97	0.0011	19.9	12.73–31.20	33.9	13.05–87.78	0.4950

**Fig. 4:**
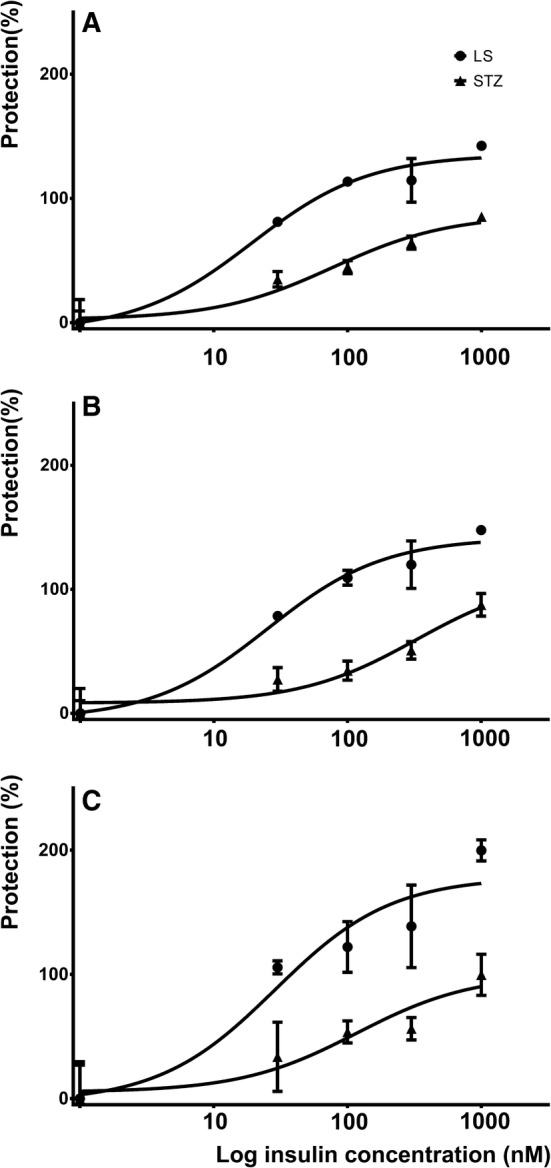
Insulin dose and time dependently improved plasma membrane integrity in LS- and 1 mM STZ-treated cells. Effect of various concentrations (30, 100, 300, 1000 nM) of insulin on LDH release of LS- and STZ-treated cells after 1, 2, and 3 days (**a**–**c**, respectively) is shown. Data are expressed as percent protection against damage

Similarly, insulin dose dependently prevented the loss of cell membrane integrity measured by LDH release assay. Insulin entirely prevented LDH release induced by LS medium, while in STZ-treated cells only an incomplete protection was observed (Fig. 4). Moreover, the concentration-response curves of insulin shifted to the right on days 1 and 2 with significant difference in the concentration inducing half maximal protective effect (Table 2).

**Table 2:** Comparison of estimates of *E*_max_ and EC50 of the protective concentration-response curve of insulin in LS- and STZ-treated cells measured by lactate dehydrogenase reduction assay

	*E*_max_					EC50				
	LS		STZ		*P*	LS		STZ		*P*
	Estimates	95% CI	Estimates	95% CI		Estimates	95% CI	Estimates	95% CI	
Day 1	135.3	123.6–147.1	86.7	70.7–102.6	0.0036	19.3	10.9–34.2	77.7	33.0–182.6	0.0205
Day 2	141.5	128.7–154.3	110.4	65.4–155.4	0.3582	25.3	14.7–43.4	326.1	90.3–1178.0	0.0019
Day 3	178.3	145.9–210.6	100.1	62.4–137.9	0.2604	28.8	10.0–82.6	117.1	23.4–584.9	0.3003

### Insulin-induced phosphorylation of GSK-3 in LS- and STZ-treated cells

**Fig. 5:**
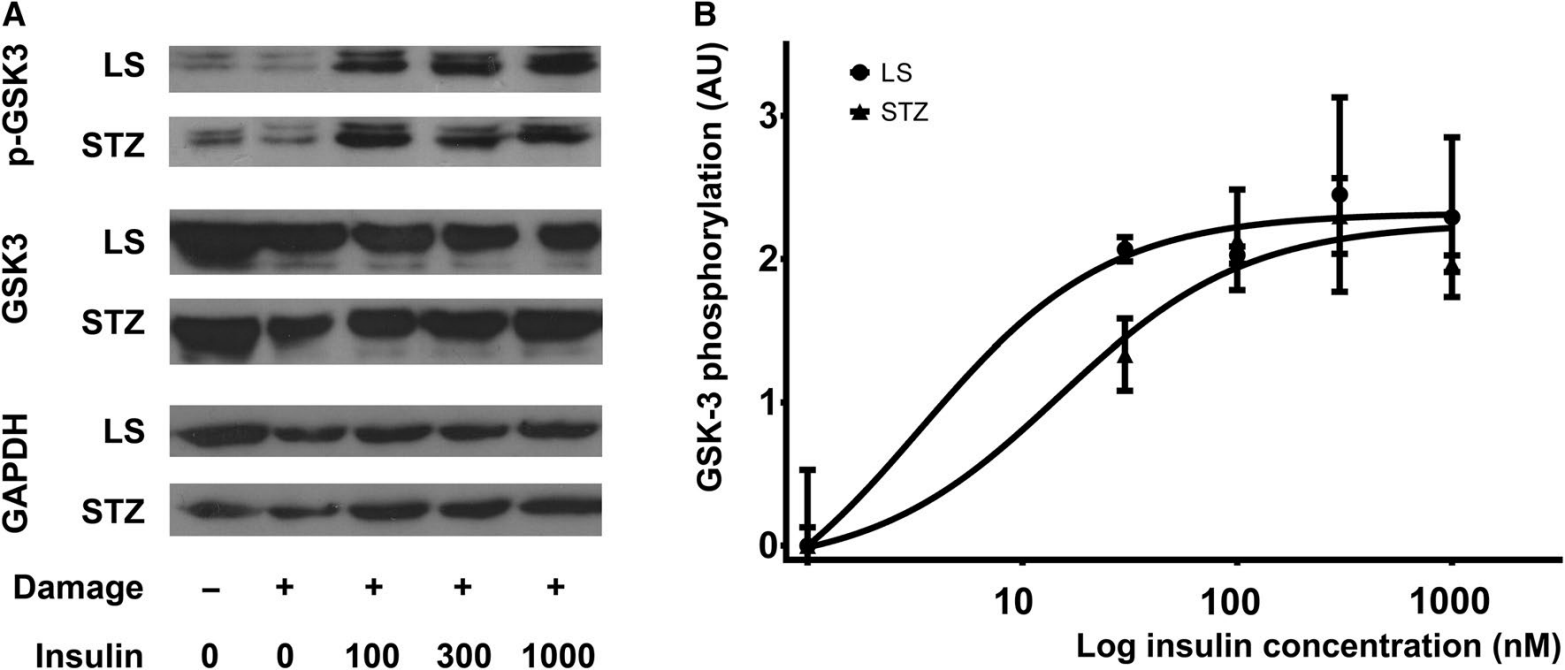
Insulin dose dependently increased GSK-3 phosphorylation in LS- and 1 mM STZ-treated cells. Effect of various concentrations (30, 100, 300, 1000 nM) of insulin on GSK-3 phosphorylation in cells cultured for 1 day in LS medium in the absence and presence of 1 mM STZ is shown. Insulin stimulation was used for 30 min before harvesting the cells. The results of western blot analysis (**a**) and ELISA measurements (**b**) are presented, *AU* arbitrary unit

Insulin-induced phosphorylation of GSK-3 was evaluated to assess the activity of insulin signaling in LS- and STZ-treated cells. Western blot analysis revealed maintained response to insulin in the STZ-treated cells. Phospho-GSK-3 levels were similar in both groups after stimulation by 100, 300, 1000 nM insulin (Fig. 5a).

For quantitation of GSK-3 phosphorylation, ELISA analysis was carried out using phospho-GSK-3 specific antibody. Insulin caused similar concentration-dependent increase in the level of phosphorylated enzyme in both LS- and STZ-treated groups (Fig. 5b). The dose-response curves were similar with no difference in neither the efficacy (*E*_max_) nor the potency (EC50) of insulin (Table 3).

**Table 3:** Comparison of estimates of *E*_max_ and EC50 of the concentration-response curve of insulin on GSK-3 phosphorylation

	*E*_max_					EC50				
	LS		STZ		*P*	LS		STZ		*P*
	Estimates	95% CI	Estimates	95% CI		Estimates	95% CI	Estimates	95% CI	
Day 1	2.32	1.97–2.66	2.25	1.90–2.60	0.7646	3.34	0.16–71.65	14.85	4.76–46.40	0.1819

## Discussion

Neurodegenerative disorders such as Alzheimer’s disease affecting the aging population are an increasing burden of the societies and there is an unmet medical need in the field of neuroprotection. The complex pathomechanism of neuronal damage is only partially elucidated and the lack of appropriate preclinical model of neurodegeneration hinders the better understanding of the diseases. Recently insulin resistance in the central nervous system was suggested to contribute to neurodegenerative processes, thus it may play a role in the development of cognitive impairment and Alzheimer’s disease (Song et al. 2015; de la Monte 2012; Schubert et al. 2004). It was previously reported that insulin signaling is compromised in the *post mortem* brain samples of Alzheimer’s disease patients (Steen et al. 2005; Freude et al. 2009; Morgen and Frolich 2015) and intranasal insulin administration (Freiherr et al. 2013; Chapman et al. 2018), as well as some other drugs like the insulin sensitizer metformin (Koenig et al. 2017; Markowicz-Piasecka et al. 2017) were shown to improve the cognitive performance. An appropriate in vitro model of neuronal insulin resistance would facilitate gaining better insight into its role in neurodegeneration and the early development of potential neuroprotective compounds.

In the present study, we assessed if there is an altered insulin effect in a previously suggested in vitro cell culture model of neurodegeneration and insulin resistance induced by STZ (Plaschke and Kopitz 2015).

Plaschke and Kopitz (2015) previously reported that 1 mM concentration of STZ induced gradual cellular damage of SK-N-MC human neuroblastoma cells assessed by LDH release. Biochemical changes characteristic for Alzheimer’s disease including accumulation of beta-amyloid peptides and activation of GSK-3 were also observed. In our present study, similar cytotoxic effect of STZ was demonstrated using the closely related SH-SY5Y neuroblastoma cells. Both resazurin reduction and LDH release tests indicated dose- and time-dependent loss of cell viability. In accordance with previous reports (Wang et al. 2011; Plaschke and Kopitz 2015), 1 mM STZ was found to cause gradually developing cytotoxicity. In some other experiments, STZ was also shown toxic on other neuronal cells and the effective concentrations were cell type specific. Biswas and coworkers used N2A mouse neuronal cell line and found it to be similarly sensitive to STZ as 1 mM concentration caused significant apoptotic cell death (Biswas et al. 2016, 2017). In primary cell cultures, however, slightly higher concentrations of STZ were found to exert similar damage. In rat cerebellar granule cells, significant cell damage was induced by 2.5–4.5 mM (Genrikhs et al. 2017) and 3–4 mM STZ (Isaev et al. 2018), while the proliferation of adult hippocampal neural stem cells (NSCs) was inhibited by 2.5 mM STZ (Sun et al. 2018). These concentrations correspond well to the expected brain level after the usual intracerebroventricular injection of 1–3 mg/kg dose (Salkovic-Petrisic et al. 2006; Grunblatt et al. 2007; Agrawal et al. 2011). This is equal to about 1–3 µmol STZ quantity that is distributed locally in a small volume of rat brain.

In addition to the cytotoxicity, altered glucose utilization was also detected in the STZ-treated cells. Decreased glucose uptake was reported in N2A neuronal and C6 astrocyte cell lines (Biswas et al. 2017) and reduced expression of glucose uptake transporters GLUT 1 and 3 was found in the same cell lines and NSCs (Biswas et al. 2017; Sun et al. 2018). Impairment in insulin signaling after STZ treatment was shown as well. In C6 astrocytes, 0.1 mM STZ decreased the insulin receptor expression and phosphorylation of IRS-1, Akt, and GSK-3. Furthermore, these alterations were completely reversed by 100 nM insulin pretreatment (Rajasekar et al. 2014). Reduced IRS-1 expression was also observed in SH-SY5Y cells treated by 0.8 mM STZ (Wang et al. 2011) and decreased GSK-3 phosphorylation was found in SK-N-MC cells after 1 mM STZ treatment (Plaschke and Kopitz 2015). Cytoprotective effect of insulin against STZ induced toxicity was also reported in cerebellar granule cells (Genrikhs et al. 2017). These results collectively suggest that STZ treatment interferes with glucose and insulin metabolism in neuronal cells; however, the development of insulin resistance was not unequivocally proven so far.

In our present experiment, the protective effect of insulin was compared in case of STZ treatment and LS condition induced loss of cell viability. As in the latter case, insulin resistance is unlikely playing a role (Deshmukh and Johnson 1997; Li et al. 2014) it can serve as control damage. LS environment caused moderate damage to SH-SY5Y cells that was further aggravated by STZ treatment in a time- and dose-dependent manner. Insulin exerted considerable concentration-dependent protective effect against both damaging conditions. Although, its maximal effect was higher in the LS group compared to STZ treated one; the concentrations causing half maximal effect in these systems were similar in most of the examined time points. These results suggest that the potency of insulin is similar in the two models indicating that no significant difference in insulin resistance (if any) was present. The slightly lower efficacy of insulin in STZ group can be explained by the more considerable damage induced by the cytotoxic compound without specific action on insulin signaling. This hypothesis was further confirmed by analyzing the insulin-induced GSK-3 phosphorylation that revealed similar effect of insulin in the STZ and LS models. GSK-3 was extensively reported to play an important role in the neurodegeneration in Alzheimer’s disease and is inactivated by insulin-induced signaling. Although, it is also regulated by other pathways, thus we cannot rule out an insulin receptor independent mechanism, thus we studied its insulin-induced phosphorylation to minimize the probability of involvement of alternative routes.

Based on our results, we can suppose that STZ in SH-SY5Y cells causes non-specific toxicity leading to gradual cell loss, however, the development of insulin resistance in this neuroblast cell line has no pivotal role in its action. Although, we found a moderate reduction in the maximal protective effect of insulin in STZ-treated cells, it is rather a consequence and not the cause of the cell damage. STZ treatment of SH-SY5Y cells thus may be an adequate in vitro model of neurodegenerative diseases, but it is not really established for studying the role of insulin resistance in neuronal death and to examine the effect of protective agents acting mainly by improving insulin signaling. However, it should be considered that we used a pure neuroblast culture that is the main limitation of our study. In in vivo experiments or co-cultures of neuronal and glial cells, a more complex mechanism of STZ might include the development of some level of insulin resistance (Gupta et al. 2018).

## Abbreviations

Akt/PKB: Protein kinase B
Aβ: Amyloid beta
DMEM/F12: Dulbecco’s Modified Eagle Medium/Nutrient Mixture F-12
FBS: Fetal bovine serum
GSK-3: Glycogen synthase kinase-3
IRS-1: Insulin receptor substrate-1
LDH: Lactate dehydrogenase
LS: Low serum
NSCs: Neural stem cells
PI3K: Phosphatidylinositol-3 kinase
STZ: Streptozotocin
TBST: Tris-buffered saline containing 0.1% Tween 20
